# Integrated Study of Canine Mammary Tumors Histopathology, Immunohistochemistry, and Cytogenetic Findings

**DOI:** 10.3390/vetsci11090409

**Published:** 2024-09-04

**Authors:** Tiago Ferreira, Maria Miranda, Rosário Pinto-Leite, João F. Mano, Rui Medeiros, Paula A. Oliveira, Adelina Gama

**Affiliations:** 1 Centre for the Research and Technology of Agro-Environmental and Biological Sciences (CITAB), University of Trás-os-Montes and Alto Douro (UTAD), 5000-801 Vila Real, Portugal; al64075@alunos.utad.pt; 2 Institute for Innovation, Capacity Building and Sustainability of Agri-Food Production (Inov4Agro), UTAD, 5000-801 Vila Real, Portugal; 3 Molecular Oncology and Viral Pathology Group, Research Center of IPO Porto (CI-IPOP)/RISE@CI-IPOP (Health Research Network), Portuguese Oncology Institute of Porto (IPO Porto), Porto Comprehensive Cancer Center (Porto.CCC), 4200-072 Porto, Portugal; ruimedei@ipoporto.min-saude.pt; 4 Department of Chemistry, CICECO—Aveiro Institute of Materials, University of Aveiro, Campus Universitário de Santiago, 3810-193 Aveiro, Portugal; jmano@ua.pt; 5 Laboratory of Genetics and Andrology, Hospital Center of Trás-os-Montes and Alto Douro, E.P.E., 5000-508 Vila Real, Portugal; mlleite@chtmad.min-saude.pt; 6 Experimental Pathology and Therapeutics Group, IPO Porto Research Center, Portuguese Institute of Oncology of Porto Francisco Gentil, E.P.E., 4200-072 Porto, Portugal; 7 Faculty of Medicine, University of Porto (FMUP), 4200-319 Porto, Portugal; 8 ICBAS—Instituto de Ciências Biomédicas Abel Salazar, Universidade do Porto, 4050-313 Porto, Portugal; 9 Molecular Oncology and Viral Pathology Group, Faculty of Health Sciences, Fernando Pessoa University, 4249-004 Porto, Portugal; 10 Research Department, Portuguese League Against Cancer (NRNorte), 4200-172 Porto, Portugal; 11 Animal and Veterinary Research Centre (CECAV), University of Trás-os-Montes and Alto Douro (UTAD), 5000-801 Vila Real, Portugal; agama@utad.pt; 12 Associate Laboratory for Animal and Veterinary Sciences (AL4AnimalS), University of Trás-os-Montes and Alto Douro (UTAD), 5000-801 Vila Real, Portugal

**Keywords:** dog, mammary tumor, age, breed, incidence, population, risk factors

## Abstract

**Simple Summary:**

Canine mammary tumors are the most common tumor in intact female dogs. This study aimed to investigate the clinicopathological, immunohistochemical, and cytogenetic features of canine mammary tumors. This study is relevant due to the fact that canine and human breast cancer share similar epidemiological and histopathological characteristics, reinforcing the potential for dogs to serve as an effective animal model for human breast cancer research.

**Abstract:**

Cancer is a complex pathological condition associated with substantial rates of mortality and morbidity in both humans and animals. Mammary gland tumors in intact female dogs are the most prevalent neoplasms. Surgical intervention remains the primary treatment choice. Alternative therapeutic options have emerged, with histopathological examination being fundamental to confirm the diagnosis and to decide the best therapy. This research focused on the clinicopathological, immunohistochemical, and cytogenetic aspects of canine mammary tumors (CMTs). Most of the animals were mixed-breed, with the majority being older than seven years, and only 16.7% had been spayed before surgery. Caudal abdominal and inguinal mammary glands were the most affected, with regional mastectomy being the predominant treatment (75.0%). Of all the tumors, 29.1% were benign, while 70.9% were malignant. Complex adenoma was the most common benign tumor, whereas tubulopapillary carcinoma was the most common malignant type. Grade III tumors (17.6%) were the least encountered, while grades I and II exhibited a similar prevalence (41.2%). All the carcinomas were classified as luminal, and cytogenetics analysis demonstrated a high chromosomal instability with significant aneuploidy observed in all cases and polyploidy detected in 62.5%. This study holds significance as canine and human breast cancers share similar characteristics, suggesting that dogs could be a valuable model for human breast cancer research. Further studies with larger sample sizes are needed to enhance our understanding of CMTs.

## 1. Introduction

Neoplasms are a prevalent concern in the canine population, particularly in older dogs, and stand as a prominent cause of death in these animals [1,2]. While advancements in veterinary medicine have expanded available treatments, accessibility to these medical resources remains a challenge for many pet owners. Among the various neoplastic conditions afflicting dogs, canine mammary tumors (CMTs) represent the second most common neoplasm, after skin tumors, and are the most frequently diagnosed neoplasia in intact female dogs [3]. Notably, mammary tumors can develop anywhere along the mammary chain, with approximately half of these cases presenting as malignant tumors [3,4]. There is no consensus in the literature as to whether pure breeds or mixed breeds are more prone to developing tumors [5,6,7,8]. However, the pure breeds that seem to develop mammary tumors more frequently are the Maltese, Yorkshire terriers, Shih Tzu, Dachshunds, Cocker spaniels, Toy poodles, and German shepherds [9]. Even in regions where preventive ovariohysterectomy procedures are widely performed, the prevalence of CMTs remains a significant concern within the field of veterinary medicine [10]. Despite the advances made in understanding CMTs, treatment options for this disease are limited when compared to the wide range of alternatives available for human breast cancer. Typically, the primary approach to managing CMTs involves surgical intervention, wherein either the tumor itself or the entire affected mammary gland is surgically excised [11].

Epidemiological studies are important in understanding the behavior of diseases, defining risk factors, and establishing fundamental prognostic criteria [9]. The role of dogs as sentinel animals is well recognized, since they share the same environment and pollutants as owners, allowing their epidemiological studies to help in the investigation of possible risk factors in the study area [12]. Furthermore, dogs are considered a good model for research into the prognosis and treatment of breast cancer, as they share several features with the human disease [13,14]. This is the first retrospective study which aims to evaluate the clinical and epidemiological data, coupled with an in-depth exploration of the immunohistopathological and cytogenetic findings associated with CMTs in Portugal.

## 2. Materials and Methods

### 2.1. Ethical Statements

The procedures were performed in accordance with European Directive (2010/63/EU). Ethical approval was granted by DGAV (Direção Geral de Agricultura e Veterinária, Lisbon, Portugal) with reference number 004582. All the participating dog owners were provided with comprehensive guidance, and the collection of clinical data and tumor samples was carried out exclusively after obtaining written consent from the owners.

### 2.2. Tumor Collection

Spontaneous CMTs were surgically excised from female dogs in veterinary hospitals or private clinics during 2021 in the northern and central regions of Portugal. Twelve female dogs were randomly selected for inclusion in this study, without any bias towards breed or age. None of the included animals had previously received anticancer treatment, and surgical intervention was undertaken based on veterinarian recommendations. Participation in the study had no effect on the clinical care provided to the animals, nor did it compromise their well-being.

Comprehensive anamnestic and clinical data were recorded for each animal, including sex, age, breed, weight, ovariohysterectomy status, contraceptive administration, and parity, as well as tumor size and location. All the data were recorded on individual sheets for each animal. For the purpose of analysis, animal weight was categorized into small (˂10 kg), medium (10 to 23 kg), and large breeds (˃23 kg), as previously defined [15]. Tumor size was divided into three distinct groups: less than 3 cm, between 3 and 5 cm, and more than 5 cm [16].

All the surgical mastectomies were performed by a veterinarian under aseptic conditions and anesthesia. The type of surgical technique performed (single mastectomy, regional mastectomy, or radical mastectomy) was also registered. Subsequent to surgical excision, fresh tissue samples were divided for further processing: one portion was immediately fixed in 10% neutral buffered formalin for histopathological diagnosis, while the other was collected for primary cell culture. In cases of multiple mammary tumors, the largest tumor was considered for the establishment of the cell culture.

### 2.3. Histopathology

The examination of tumor samples was conducted at the Histopathology Laboratory at the University of Trás-os-Montes and Alto Douro (UTAD, Vila Real, Portugal). After at least 24 h of fixation in formalin, the samples were processed for routine histopathology. Three-micrometer-thick sections were cut and stained with hematoxylin and eosin (H&E).

Histopathological analysis was carried out by an experienced veterinary pathologist (Adelina Gama) using a light microscope (Nikon Eclipse E6000^®^, Nikon Instruments Inc., Melville, NY, USA) with a digital camera. The mammary tumors were classified histologically using the most recently proposed classification system [17]. The presence of intra-tumoral necrosis was assessed in each neoplastic lesion. Inflammatory infiltrate was also assessed and classified as absent, slight, moderate, and marked. Furthermore, vascular invasion was assessed for each malignant lesion. Lymph node metastasis was assessed in the cases where lymph nodes were present (*n* = 7).

For all the carcinomas, histological grade was determined according to the classification system established by [18], considering three morphological features: (1) tubule formation, (2) nuclear pleomorphism, and (3) mitotic count. Grade I indicates a well-differentiated or low-grade carcinoma; grade II denotes a moderately differentiated or intermediate-grade carcinoma; and grade III represents a poorly differentiated or high-grade carcinoma.

### 2.4. Immunohistochemistry

Immunohistochemical staining was performed on the carcinoma samples specifically collected for cell culture, using the Novolink^TM^ Max Polymer Detection System Kit (RE7280-K, Leica Biosystems, Newcastle, UK), according to the manufacturer’s instructions. As described in Table 1, the tissue sections were incubated with the following biomarkers: monoclonal mouse anti-human estrogen receptor alpha (ERα), monoclonal mouse anti-human progesterone receptor (PR), polyclonal rabbit anti-human c-erbB-2 oncoprotein (HER2), and monoclonal mouse anti-human ki-67. The sections were deparaffinized in xylene, followed by rehydration using decreasing concentrations of alcohol and a final rinse in distilled water. Antigen retrieval was achieved by heating the slides in a 0.01 mol/L citrate buffer (pH 6.0) in the microwave (3 cycles of 5 min, 750 W, including stirring). Subsequently, endogenous peroxidase activity was blocked for 20 min using 0.3% hydrogen peroxide, followed by protein blocking for 5 min (for PR and ki-67 antibodies) or 10 min (for ER and HER2). After overnight incubation with primary antibodies at 4 °C, the slides underwent three washes with phosphate-buffered saline (PBS). Afterwards, the sections were incubated with a post-primary solution for 30 min and rinsed with PBS, followed by a 30 min incubation with the polymer. The antigen–antibody complex was visualized by incubating the sections in 3,3′-diaminobenzidine (DAB) and washed in tap water (10 min). The slides were counterstained with hematoxylin for 2 min, then dehydrated through an ethanol series and mounted with Entellan^®^. Finally, the prepared slides were air-dried and stored until light microscopy analysis.

### 2.5. Evaluation of Immunohistochemical Data

The slides stained with ERα, PR, and HER2 antibodies were analyzed by the same experienced veterinary pathologist. The immunoexpression of ERα and PR was assessed using a semi-quantitative approach based on the Allred scoring system, as previously described [18]. In brief, the total score is calculated as the sum of the percentage of stained cells and the intensity of immunolabeling. The total score ranged from 0 to 8, with a threshold of ≥3 being used to classify tumors as ER/PR-positive. Immunoreactivity to HER2 was scored according to [18]. The scores 0, 1+, and 2+ are classified as negative, while a score of 3+ is defined as positive. The Ki-67 index was determined by counting the number of Ki-67-positive cells per 1000 cells, sampled from five random HPFs (400× magnification), by using the QuPath software (Quantitative Pathology & Bioimage Analysis, v0.3.2 for Windows) on the digital images. The ki-67 index was classified as high or low based on the median obtained.

Molecular subtypes were assigned based on the following criteria: luminal A-like (ER- and/or PR-positive, HER2-negative and low Ki-67); luminal B-like (HER2-negative) (ER- and/or PR-positive, HER2-negative and high Ki-67); luminal B-like (HER2-positive) (ER- and/or PR-positive, HER2-positive, any Ki-67); HER2-positive (non-luminal) (ER- and PR-negative and HER2-positive); and triple-negative (ER-, PR-, and HER2-negative), according to) [19].

### 2.6. Follow-Up Data

The female dogs included in this study were monitored post-surgically for a period of three years. A comprehensive investigation into tumor recurrence, systemic metastases, and overall survival status was conducted through direct contact with the referring veterinarian. In cases where the animals presented multiple carcinomas, the higher-grade lesion was prioritized for analysis.

### 2.7. Primary Cell Culture and Karyotype Analysis

Under sterile conditions, the 12 tumor samples were minced into small fragments and then transferred into microtubes. Next, 1.5 mL of 0.25% trypsin-EDTA (Biological Industries, Kibbutz Beit Haemek, Israel) were added to the microtube and allowed to act for 30 min. Following this, the mixture was centrifugated at 1200 rpm for 10 min. Subsequently, 1.5 mL of 0.02% collagenase type IA (Gibco, Life Technologies, Bleiswijk, The Netherlands) was applied to the disaggregated cells for another 30 min. Following the completion of the digestion process, the cells were transferred into 25 cm^2^ sterile flasks (Orange Scientific, Braine-l’Alleud, Belgium). Subsequently, each flask was supplemented with 5 mL of RPMI 1640 culture medium (PAN Biotech, Aldenbach, Germany) supplemented with 10% fetal bovine serum (FBS) (PAN Biotech, Aldenbach, Germany), 100 U/mL penicillin (Biological Industries, Kibbutz Beit Haemek, Israel), 100 μg/mL streptomycin (Biological Industries, Kibbutz Beit Haemek, Israel), and 2 mM L-Glutamine (Sigma Aldrich, St. Louis, MO, USA). The cells were then incubated at 37 °C in a humidified atmosphere containing 5% CO_2_.

Upon the successful establishment of the cell culture, the number of chromosomes was determined by karyotype. Briefly, the cells were treated with 10 μL/mL colcemid (Biological Industries, Kibbutz Beit Haemek, Israel) for 3 h. Subsequently, the cells were treated with trypsin-EDTA and subjected to a hypotonic treatment (8:1 of 0.05 M potassium chloride: FBS) at 37 °C for 20 min. The freshly prepared fixative solution, consisting of methanol and acetic acid (3:1), was then used and washed three times. The resulting suspension was dropped on a microscope slide, and the standard trypsin–Leishman (GTL-banding) method was performed. The metaphases were digitally captured using an automatic microscope (Leica DM6000 B, Leica Microsystems GmbH, Wetzlar, Germany) and a total of 50 metaphases with well-spread chromosomes were evaluated.

### 2.8. Statistical Analysis

A descriptive statistical analysis was performed, with data presented as mean values ± standard deviation. Statistical analysis was performed using GraphPad Prism^®^ (v9.5.0) software for Windows (GraphPad Software Inc., La Jolla, CA, USA), and *p* values less than 0.05 (*p* < 0.05) were considered as statistically significant. Associations between categorical variables (between histological type and grade, and histological type and immunophenotype) were performed using the Fisher exact test. For histological type categorization, the carcinomas were grouped into simple (tubulopapillary and solid carcinomas) and non-simple (carcinomas in a complex adenoma, complex carcinomas, carcinoma arising in a complex adenoma/benign mixed tumor, and mixed carcinoma) carcinomas.

## 3. Results

### 3.1. Clinicopathological Features

This study encompassed a cohort of twelve female dogs that underwent surgical excision of their CMTs. The tumor samples were collected from female dogs of different breeds, predominantly mixed-breed (*n* = 6; 50.0%), with an average age of 8.2 ± 3.0 years [range (4–15), median 7 years] at the time of surgical tumor removal. The majority of cases (*n* = 8; 66.7%) were between 7 and 15 years. Among the ten female dogs for which weight information was available, five (50.0%) weighed more than 23 kg (average 18.7 ± 9.7 weight [range (4.3–30), median 30.0 kg]). Most of the animals were intact (*n* = 7; 58.3%), and only one case (*n* = 1; 8.3%) had a confirmed history of contraception administration. Parity information was unavailable for five cases (41.7%).

Out of the twelve female dogs, a total of 17 tumors were identified, with the majority affecting the inguinal mammary glands (*n* = 7; 41.2%) and the left mammary chain (*n* = 10, 58.8%). The mean tumor size measured 3.3 ± 2.6 cm, with a median of 2.4 cm. The most commonly performed surgical procedure was regional mastectomy, involving the excision of the two or more mammary glands (*n* = 9; 75.0%). The detailed epidemiological data collected through the questionnaire are described in Table 2.

In terms of histological classification, twenty-four lesions, comprising seven benign (29.1%) and seventeen malignant (70.9%) tumors, were observed in a cohort of twelve female dogs; detailed information is given in Table 3. Regarding malignant neoplasms, nine (53.0%) were classified as simple carcinomas, while seven (41.2%) were categorized as non-simple carcinomas. Additionally, one case was classified as a special type (5.8%) (Table 3 and Supplementary Figure S1).

Regarding neoplastic lesions, necrosis was identified in five cases (20.8%), and the majority of cases exhibited an absence of inflammatory infiltrate (*n* = 13; 54.1%), as shown in Table 4. Vascular invasion was present in three carcinomas (17.6%). Of the seven cases with available lymph nodes, three (42.9%) presented lymph node metastases. Using the grading system established by Peña (2013), the invasive carcinomas were classified as grade I (*n* = 7; 41.2%), grade II (*n* = 7; 41.2%), and grade III (*n* = 3; 17.6%), as detailed in Table 4.

### 3.2. Immunohistochemistry

Among the 12 carcinomas analyzed for immunophenotype identification, all the cases expressed ER, while only seven samples (58.3%) were positive for PR (Supplementary Figure S2). The median Ki-67 index was found to be 20.9% [range (8.2–45.1%); mean 22.9 ± 11.2%]. Six cases (50.0%) exhibited high proliferation (Ki-67 index > 20.9%). Only the grade III tumors were classified as high proliferation types, whereas the grade I and II tumors were categorized as those showing both high and low proliferation. Luminal A-like and luminal B-like (HER2-negative) subtypes were observed in equal proportions (*n* = 6; 50.0%), as indicated in Table 5. No carcinoma was classified as HER2-positive. Thus, no case was classified as a luminal B-like (HER2-positive), HER2-positive (non-luminal), or triple-negative subtype.

After a 3-year follow-up period, one animal was excluded due to insufficient follow-up data. One animal died (case 4) after 11 months of surgery. The remaining animals (*n* = 10; 90.9%) were alive without tumor relapse/metastatic disease.

Table 6 presents an overview of the canine mammary carcinomas included in this study, considering clinical, histopathological, and immunohistochemical characteristics.

### 3.3. Relationship between Histological Type, Grade, and Immunophenotype

To analyze the association between the histological type and the grade or immunophenotype, the carcinomas were grouped into simple and non-simple carcinomas. In Table 7 and Table 8, the relationships between histological type and grade, and between histological type and immunophenotype, respectively, are described. No differences were found in any of the cases.

### 3.4. Karyotype Analysis

**Figure 1:**
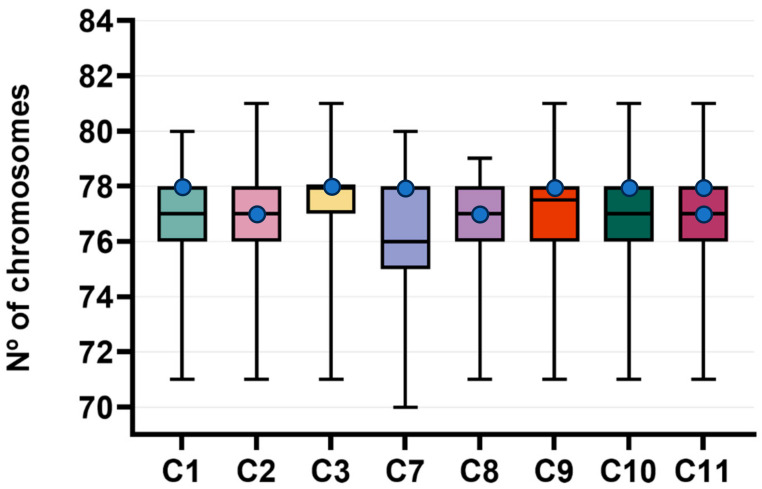
Chromosome numbers per canine mammary tumor cell line. The chromosome number deviated abnormally from the normal canine diploid number (2*n* = 78), indicating a variety of aneuploidy in all primary cell cultures. Blue dots represent modal value for each primary cell culture. C: case.

Of the 12 samples used, eight were successfully established in the primary cell culture (cases 1–3, 7–11), and fifty metaphases were obtained per culture. In all the cases, hypoploid, hyperploid, and diploid metaphases were observed, with the chromosome count ranging from 70 to 81. Aneuploidy was present in all the cases, with case 11 showing the highest percentage of aneuploidy (78%), of which 60% was hypoploid and 18% hyperploid. Case 3 had the highest frequency of diploid cells (44%) (Table 9), and in the remaining cases, the metaphases had a median chromosome number below the normal diploid number (2n = 78) (Figure 1). The modal number of 78 was observed in cases 1, 3, 7, 9, and 10; 77 was observed in cases 2 and 8, while case 11 revealed a bimodal value of 77 and 78 chromosomes. Regarding tumor grade, both cases 3 and 11 were grade II, while case 7 was grade III, presenting the second highest frequency of hypoploid, 72%, and the lowest frequency of hyperploidy, 2%. Polyploidy (with chromosome counts ranging from 142 to 157 chromosomes) was observed in 10 metaphases of grade I, II, and III tumors (cases 2, 7, 8, 9, and 11), with grade II tumors accounting for three of the five cases with polyploidy.

## 4. Discussion

CMTs hold substantial significance in veterinary medicine; this is particularly due to the increasing lifespan of animals. They are prevalent among females, and understanding the prognosis of these neoplasms is crucial for determining the most appropriate treatment. This study focused on spontaneous mammary tumors obtained from female dogs referred for examination and treatment by veterinary surgeons, where surgery was the only treatment carried out.

Mixed breeds were the prominent dog breed affected; however, the association between breed and incidence is limited by our small sample size. However, previous studies have shown that mixed-breed dogs have a higher incidence of mammary tumors [5,6]. Conversely, some studies propose that pure breeds are the most susceptible to this disease [8,12,20]. The controversy regarding breed predisposition in the literature might be attributed to variations in the profile of the canine population under study. In fact, one study indicates that the incidence of CMTs varies according to breed, but the breeds considered to be at risk differ in different studies and in different geographic locations [21].

The frequency of mammary tumors exhibited an association with age, being less frequent in female dogs younger than 6 years but increasing up to 66.7% for those between 7 and 15 years. A recent study with 92 cases also highlighted age as a significant risk factor, reporting an average occurrence age ranging between 8 and 13 years [22]. Malignant tumors are rare before the age of 5 [7,20]. However, we identified a malignant tumor (carcinoma arising in a complex adenoma) in a four-year-old purebred French bulldog (case 2). The majority of tumors were predominantly found in the inguinal mammary gland (M5, 41.2%), as observed by other authors [23,24,25]; this is potentially due to the larger amount of glandular tissue present [26]. While several studies indicate that most of the tumors found were single nodules [8,23], we observed an equal number of dogs with single and multiple nodules. Interestingly, 60.0% of the tumors were smaller than 3 cm, with regional mastectomy being the most common treatment option (*n* = 9, 75.0%). This is in accordance with the established notion that small tumors generally have a more favorable prognosis than larger ones [7,27]. In fact, lesions larger than 5 cm are associated with malignancy, higher rates of cell proliferation, and lower expression of hormone receptors [28]. Given the relevance of tumor size, measuring it is a simple and low-cost standard method, helping in the surgery type selection for each individualized patient and in prognosis prediction.

Most of the female dogs involved in this study were not sterilized, aligning with findings suggesting that OVH offers a protective effect against mammary tumor development, likely through sex steroid inhibition [12,29,30,31]. Notably, 74% of malignant tumors were identified in intact dogs, diagnosed at a significantly younger age compared to spayed dogs (9.20 vs. 10.09 years, respectively) [12]. Our study noted only one animal under hormonal treatment, linking hormone exposure to increased CMT risk, particularly with steroid hormones such as 17β-estradiol (E2), which promote cell proliferation and act as an anti-apoptotic agent in tumor growth [32].

Among the identified neoplasms, 7 (29.1%) were classified as benign, while 17 (70.9%) were classified as malignant, aligning with previous studies that also reported a higher incidence of malignant tumors [12,33]. This difference may stem from the increased detectability of malignant tumors compared to benign ones and a treatment bias toward addressing more phenotypically concerning tumors. Essentially, as they are seemingly benign, small and slow-growing tumors might not always be excised, resulting in a lower number of identified benign tumors. Among the benign tumors, the majority consisted of complex adenomas (*n* = 3; 42.3%), a pattern which is consistent with the findings of Vascellari and colleagues [12]. Conversely, among the malignant tumors, simple tubulopapillary lesions were the most common type (*n* = 8; 47.1%), which is in line with the observations made by Burrai and co-workers [33]. Our study noted a higher percentage of tumors with ERα (100.0%) expression compared to PR (58.3%). The Ki-67 index analysis revealed an equal number of cases classified as those showing high and low proliferation. Specifically, grade III tumors were exclusively categorized as highly proliferative, aligning with the association of a high Ki-67 index with poor prognosis in malignant CMTs [34,35]. Notably, we observed histological grade III tumors in two older animals (11 and 15 years old), corroborating previous studies that established a link between age and tumor malignancy and grade [6,7,12]. With regard to the molecular classification of carcinomas, our study classified them as luminal, with no observations of HER2-positive or triple-negative subtypes. The presence of hormonal receptors within these malignant tumors suggests a more favorable prognosis compared to the HER2-positive and triple-negative subtypes, which are associated with poor prognosis [16,36]. No HER2-positive cases (defined by a score of 3 + by HER2 immunohistochemistry) were detected in our study, which is in line with other studies where this immunophenotype was either not found [36] or found in a low percentage (8.3% and 9.1%) [16,37]. No triple-negative immunophenotype was found either. However, there is no consensus in the literature on this immunophenotype, as its prevalence is highly variable, with some studies showing a high prevalence (69.0% and 76.3%) [36,38] and others showing a low prevalence (14.7% and 15.4%) [37,39]. In fact, the majority of the female dogs included in the present study have long disease-free survival times, with only one case associated with death due to mammary carcinoma metastatic disease. This female dog presented an invasive solid carcinoma, grade III, with a luminal B phenotype. This subtype is traditionally associated with high proliferation and an aggressive behavior, when compared to luminal A phenotype, in both dogs and humans [36,40,41].

Canine karyotyping is a difficult procedure due to the high number of chromosomes (2*n* = 78), with all the autosomes having acrocentric morphology and only the sex chromosomes being biarmed [42]. Most chromosomes are small, and their banding patterns do not allow all homologues to be recognized unequivocally [42]. Therefore, and to ensure a non-biased analysis, we opted, like other studies, to examine the chromosome count. Karyotypic features support that the samples were of *C. familiaris* origin, as normal diploid metaphases were present in all the tumors. The tumoral progression is associated with chromosomal instability, which may evolve and lead to the appearance of polyploidies [43,44,45]. In all the tumors, we detected chromosomal instability, as the percentage of aneuploidy ranged from 56 to 78% in the various samples. Our results are in accordance with those of a previous study that analyzed the chromosome count in a HER2-overexpressing canine myoepithelial cancer cell line and revealed that all the cells exhibited a lower than normal number of chromosomes (ranging from 31 to 64 chromosomes) [46]. Regarding aneuploidy, another study also examined the chromosome count of 10 canine mammary cancer cell lines and detected both hypo- and hyperploid cell lines, as in our findings [47]. Moreover, the analysis of two triple-negative canine mammary cancer cell lines, one originating from an inflammatory mammary carcinoma and another from an intraductal papillary carcinoma, unveiled a modal number of 78 chromosomes [48,49]. In our sample, 62.5% had polyploidy in grade I, II and III, which may indicate high chromosomal instability regardless of the type of grade. In fact, elevated aneuploidy is correlated with high tumor grade, poor prognosis, and metastasis [50].

Although this study contributes to the existing body of knowledge regarding CMTs, several limitations should be acknowledged. As mentioned above, the relatively small sample size limits the generalizability of the findings. Yet, this study has the advantage of being a prospective study, not only presenting the clinicopathological characterization, but also integrating its molecular phenotype identification and the karyotype analysis.

## 5. Conclusions

This study is a prospective study of canine mammary tumors, integrating clinicopathological characterization, follow-up data, molecular phenotype classification, and cytogenetic analysis. Although the study included a relatively small sample size, age stands out as a critical risk factor for CMTs, but the absence of a clear breed-related association highlights the intricate interplay of genetic and environmental factors in this disease’s development. Most of the analyzed carcinomas were classified as histological grade I or II and as having a luminal phenotype, with a predominance of luminal A, which are characteristics associated with a favorable prognosis. Cytogenetic analysis revealed the prevalence of aneuploidy and polyploidy in a substantial portion of the CMT cell lines, emphasizing the genetic complexity underlying these tumors. Comprehensive genetic profiling is crucial for improved prognostic and personalized treatment approaches. Larger sample sizes and extended follow-up periods are also necessary to assess long-term outcomes and survival rates, aiding the development of more effective therapeutic strategies and prognostic tools for canine mammary tumors.

## Figures and Tables

**Table 1:** Primary antibodies and immunostaining protocols used.

Antibody	Clone (Code Number)	Origin	Dilution	Pre-Treatment	Incubation
ERα	C-311 (sc-787)	Santa Cruz Biotechnology, Dallas, TX, USA	1:100	Microwave	Overnight
PR	NCL-L-PGR (312)	Novocastra, UK	1:40	Microwave	Overnight
HER2	Polyclonal (A0485)	Dako, Denmark	1:40	Microwave	Overnight
Ki-67	MIB-1 (M7240)	Dako, Denmark	1:50	Microwave	Overnight

**Table 2:** Clinicopathological characteristics of the dogs and tumors included in the present study.

	N	%
Breed (*n* = 12)		
Mixed	6	50.0
Labrador Retriever	2	16.8
Boxer	1	8.3
French Bulldog	1	8.3
German Shepherd	1	8.3
Pinscher	1	8.3
Age (years; *n* = 12)		
7 years	8	66.7
>7 years	4	33.3
Weight (kg; *n* = 10) ^a^		
<10 kg	4	40.0
10–23 kg	1	10.0
>23 kg	5	50.0
Ovariohysterectomy (*n* = 12)		
No	7	58.3
Yes, prior to tumor development	2	16.7
Yes, performed with mastectomy	3	25.0
Contraception (*n* = 12)		
No	11	91.7
Yes	1	8.3
Parity (*n* = 12)		
Nulliparous	4	33.3
Primiparous	2	16.7
Multiparous	1	8.3
Unknown	5	41.7
Multicentricity (*n* = 17)		
Single	6	50.0
Multiple	6	50.0
Tumor location (*n* = 17)		
Thoracic glands (M1–M2)	4	23.5
Abdominal glands (M3–M4)	6	35.3
Inguinal glands (M5)	7	41.2
Mammary chain (*n* = 17)		
Left	10	58.8
Right	7	41.2
Tumor size (*n* = 17)		
<3 cm	11	64.7
3–5 cm	2	11.8
>5 cm	4	23.5
Surgical procedure (*n* = 12)		
Single mastectomy	3	25.0
Regional mastectomy	9	75.0

^a^ Weight was available in ten cases.

**Table 3:** Histological diagnosis of canine mammary tumors.

		N	%
Benign epithelial neoplasms			
Simple benign tumors	Adenoma—simple	1	4.2
Non-simple benign tumors	Complex adenoma	3	12.5
Ductal associated benign tumors	Intraductal papillary adenoma	2	8.3
	Ductal adenoma	1	4.2
Malignant epithelial neoplasms			
Simple carcinoma	Tubulopapillary carcinoma	8	33.3
	Solid carcinoma	1	4.2
Non-simple carcinoma	Carcinoma arising in a complex adenoma/benign mixed tumor	2	8.3
	Complex carcinoma	2	8.3
	Carcinoma and malignant myoepithelioma	1	4.2
	Mixed carcinoma	2	8.3
Special Type	Adenosquamous carcinoma	1	4.2
Total		24	100

**Table 4:** Clinicopathological features of canine mammary tumors.

	N	%
Necrosis (*n* = 24)		
Absent	19	79.2
Present	5	20.8
Inflammatory infiltrate (*n* = 24)		
Absent	13	54.1
Slight	0	0.0
Moderate	4	16.7
Marked	7	29.2
Vascular invasion (*n* = 17)		
Absent	14	82.4%
Present	3	17.6%
Lymph node metastasis (*n* = 7) ^a^		
Absent	4	57.1
Present	3	42.9
Histological grade (*n* = 17)		
Grade I	7	41.2
Grade II	7	41.2
Grade III	3	17.6

^a^ Lymph nodes were available in seven cases.

**Table 5:** Frequencies of the immunohistochemical biomarkers and immunophenotypes of canine mammary carcinomas.

	N	%
ERα (*n* = 12)		
ER+	12	100
ER−	0	0
PR (*n* = 12)		
PR+	7	58.3
PR−	5	41.7
HER2 (*n* = 12)		
0	4	33.3
1+	6	50.0
2+	2	16.7
3+	0	0.0
Ki-67 (*n* = 12)		
Ki-67 low (≤20.9%)	6	50.0
Ki-67 high (>20.9%)	6	50.0
Subtype (*n* = 12)		
Luminal A-like (Ki-67 index ≤ 20.9%)	6	50.0
Luminal B-like (HER2-negative) (Ki-67 index > 20.9%)	6	50.0
Luminal B-like (HER2-positive)	0	0.0
HER2-positive	0	0.0
Triple-negative	0	0.0

**Table 6:** Overview of carcinomas included in the present study.

Case #	Breed	Gender	Age	Histology	Tumor Grade	Molecular Subtype
1	Mixed	♀	10	Complex carcinoma	I	Luminal B-like (HER2-negative)
2	French Bulldog	♀	4	Carcinoma arising in a complex adenoma	I	Luminal A-like
3	Mixed	♀	7	Carcinoma-and-malignant myoepithelioma	II	Luminal A-like
4	Mixed	♀	15	Solid carcinoma	III	Luminal B-like (HER2-negative)
5	Labrador retriever	♀	7	Tubulopapillary carcinoma	II	Luminal A-like
6	Mixed	♀	6	Tubulopapillary carcinoma	I	Luminal A-like
7	Mixed	♀/n	11	Tubulopapillary carcinoma	III	Luminal B-like (HER2-negative)
8	Mixed	♀	6	Tubulopapillary carcinoma	II	Luminal B-like (HER2-negative)
9	Pinscher	♀	6	Complex carcinoma	II	Luminal A-like
10	Boxer	♀	7	Tubulopapillary carcinoma	I	Luminal A-like
11	Labrador	♀/n	7	Mixed carcinoma	II	Luminal B-like (HER2-negative)
12	German shepherd	♀	8	Mixed carcinoma	I	Luminal B-like (HER2-negative)

Case # = case number as referred to within this study; ♀ = female, intact; ♀/n = female, neutered; age = patient age at excision of tumor.

**Table 7:** Association between histological type and grade.

Histological Type	Histological Grade		*p*
	Grade I	Grade II/III	
Simple carcinoma	2 (13.3%)	7 (46.7%)	0.329
Non-simple carcinoma	3 (20.0%)	3 (20.0%)	
Total	5 (33.35)	10 (66.7%)	15 (100.0%)

**Table 8:** Association between histological type and immunophenotype.

Histological Type	Immunophenotype		*p*
	Luminal A-Like	Luminal B-Like (HER2-Negative)	
Simple carcinoma	3 (25.0%)	3 (25.0%)	0.999
Non-simple carcinoma	3 (25.0%)	3 (25.0%)	
Total	6 (50.0%)	6 (50.0%)	12 (100.0%)

**Table 9:** Distribution of aneuploidy and polyploidy, and tumor grade in each case.

Case #	Tumor Grade	Diploidy (%)	Aneuploidy (%) (%Hypoploidy + %Hyperploidy)	Number of Polyploidy
1	I	34.0	66.0 (58.0 + 8.0)	*–*
2	I	28.0	72.0 (56.0 + 16.0)	2
3	II	44.0	56.0 (40.0 + 16.0)	*–*
7	III	26.0	74.0 (72.0 + 2.0)	1
8	II	24.0	76.0 (68.0 + 8.0)	2
9	II	38.0	62.0 (50.0 + 12.0)	2
10	I	36.0	64.0 (52.0 + 12.0)	*–*
11	II	22.0	78.0 (60.0 + 18.0)	3

## Data Availability

The data are contained in the manuscript and Supplementary Materials.

## Supplementary Materials

The following supporting information can be downloaded at: https://www.mdpi.com/article/10.3390/vetsci11090409/s1, Figure S1: Histopathological analysis of canine mammary tumors. (A) Tubulopapillary carcinoma; (B) Carcinoma-and-malignant myoepithelioma; (C) Solid carcinoma, high grade and (D) Lymph node metastasis of the tubulopapillary carcinoma. H&E staining. Figure S2: Immunohistochemical expression of canine mammary carcinomas: carcinomas positive for (**A**) ER, (**B**) PR, (**C**) HER2 and (**D**) ki-67. Counterstained with hematoxylin.
