# Supplementary material for: Integrated Study of Canine Mammary Tumors Histopathology, Immunohistochemistry, and Cytogenetic Findings
Source: Vet Sci. 2024 Sep 4;11(9):409. doi: 10.3390/vetsci11090409 (PMC11435489; doi:10.3390/vetsci11090409)
Supplement: Supplementary file 1 [file vetsci-11-00409-s001.zip › vetsci-3136134-supplementary.pdf]

Integrated study of canine mammary tumors histopathology, immunohistochemistry, and cytogenetic findings

Supplementary Material

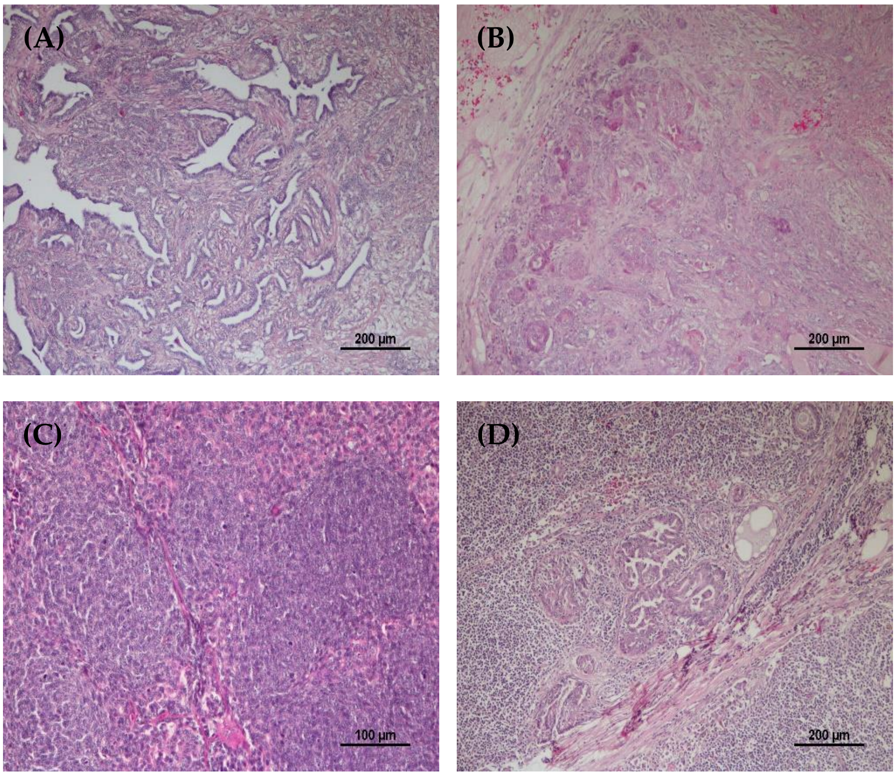

Figure S1. Histopathological analysis of canine mammary tumors. (**A**) Tubulopapillary carcinoma; (**B**) Carcinoma-and-malignant myoepithelioma; (**C**) Solid carcinoma, high grade and (**D**) Lymph node metastasis of the tubulopapillary carcinoma. H&E staining.

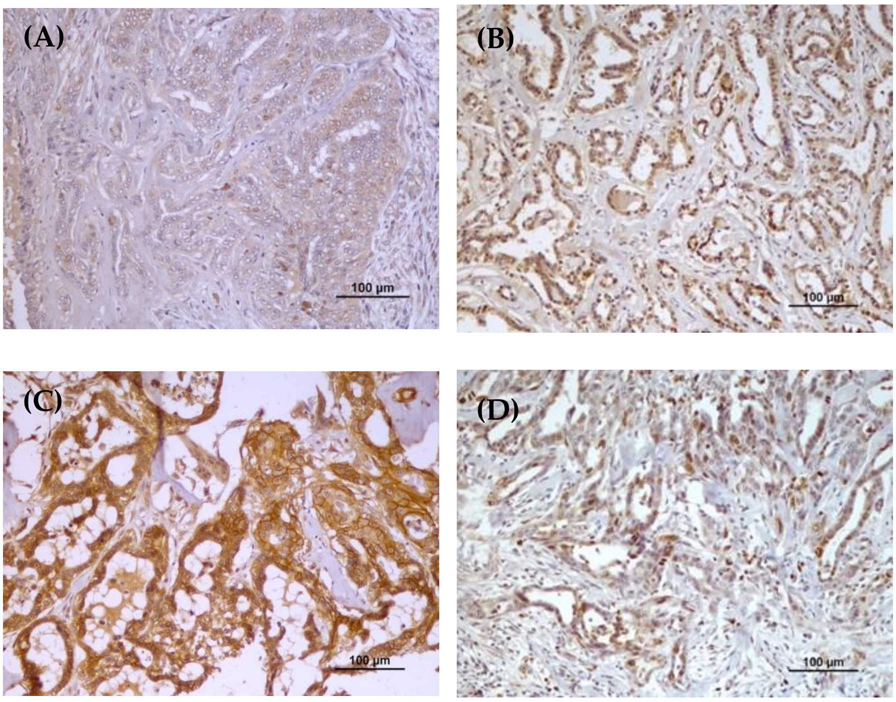

Figure S2. Immunohistochemical expression of canine mammary carcinomas: carcinomas positive for (**A**) ER, (**B**) PR, (**C**) HER2 and (**D**) ki-67. Counterstained with hematoxylin.
